# Pupil-Linked Arousal Determines Variability in Perceptual Decision Making

**DOI:** 10.1371/journal.pcbi.1003854

**Published:** 2014-09-18

**Authors:** Peter R. Murphy, Joachim Vandekerckhove, Sander Nieuwenhuis

**Affiliations:** 1 Department of Psychology and Leiden Institute for Brain and Cognition, Leiden University, Leiden, The Netherlands; 2 Department of Cognitive Sciences, University of California, Irvine, Irvine, California, United States of America; Oxford University, United Kingdom

## Abstract

Decision making between several alternatives is thought to involve the gradual accumulation of evidence in favor of each available choice. This process is profoundly variable even for nominally identical stimuli, yet the neuro-cognitive substrates that determine the magnitude of this variability are poorly understood. Here, we demonstrate that arousal state is a powerful determinant of variability in perceptual decision making. We measured pupil size, a highly sensitive index of arousal, while human subjects performed a motion-discrimination task, and decomposed task behavior into latent decision making parameters using an established computational model of the decision process. In direct contrast to previous theoretical accounts specifying a role for arousal in several discrete aspects of decision making, we found that pupil diameter was uniquely related to a model parameter representing variability in the rate of decision evidence accumulation: Periods of increased pupil size, reflecting heightened arousal, were characterized by greater variability in accumulation rate. Pupil diameter also correlated trial-by-trial with specific patterns of behavior that collectively are diagnostic of changing accumulation rate variability, and explained substantial individual differences in this computational quantity. These findings provide a uniquely clear account of how arousal state impacts decision making, and may point to a relationship between pupil-linked neuromodulation and behavioral variability. They also pave the way for future studies aimed at augmenting the precision with which people make decisions.

## Author Summary

Variability is a hallmark of how we make decisions between different alternatives: Even when we are presented with identical repetitions of a stimulus, the timing and accuracy of our associated decisions vary dramatically. Representations of variability or ‘noise’ have necessarily been a prominent feature of how cognitive scientists model the decision making process. However, very little is known about the underlying neural processes or psychophysiological states that determine the magnitude of this variability. In this study, we measured people's pupil size as an indicator of their physiological arousal state during performance of a challenging motion-discrimination task, and modelled decisions on this task using an established computational model of the decision process in which evidence gradually accumulates toward a response threshold. We found that arousal state was tightly and uniquely linked to a computational parameter that specifically represents variability in the rate at which people accumulate evidence to inform their decisions: Larger pupil size, both within- and between-individuals, corresponded to greater variability in this critical aspect of decision making. Our findings uncover a potent source of variability in how people make decisions, and forge a new link between the classical construct of arousal and modern theories of decision making.

## Introduction

Decisions that must be made between two or more alternatives are faced throughout everyday life. Such decisions are thought to involve a sequential process whereby evidence is gradually accumulated in favor of each choice option, until a threshold level corresponding to a specific choice is reached [1]–[4]. Adjustments to discrete elements of this accumulation-to-bound process can be strategically deployed to optimize decision making in a given context [1], [5]–[7]. Yet even when the strategic demands of a task are held constant, the timing and accuracy of decisions are profoundly variable. While recent research has worked to characterize the neural correlates of trial-to-trial variability in decision making [8]–[14], very little is known about the neuro-cognitive processes that actually determine the magnitude of this variability. Characterizing sources of such variability is thus a key challenge for the cognitive neuroscience of decision making.

Here, we focus on changes in arousal state as a primary source of variability in perceptual decision making. Arousal broadly refers to an organism's state of responsivity to external stimulation [15], and is a principal determinant of the manner in which organisms engage with their environments. Intermediate levels of arousal promote focused engagement in the task at hand, whereas departures from this ‘optimal’ arousal state can lead to drowsiness and demotivated behavior at one extreme, or distractibility at the other extreme [16]–[19]. Thus arousal exhibits an ‘inverted-U’ relationship with task engagement, suggesting that the capacity for optimal decision making may decline at overly low or high arousal levels. Arousal state is regulated by the tonic activity of the brain's neuromodulatory systems [16], [20]–[22], which exert potent influence over the dynamics of neural activity (e.g. by modulating the responsivity or ‘gain’ of neural networks [16], [23]–[25]). Spontaneous, task-independent fluctuations in neuromodulatory tone have been strongly associated with decreased sensitivity to task-relevant stimuli and with more variable and erratic responding in ways that mirror the non-linear relationship between arousal state and task engagement [16], [25], [26].

Several mechanistically explicit accounts have attempted to link changes in arousal and their neuromodulatory foundations to overt behavioral sequelae in a number of specific contexts ([e.g. simple target-detection; [16], [24], [25], [27]). However, the relationship between arousal state and the basic computations underlying accumulation-to-bound decision making is still poorly understood. Arousal and neuromodulation might plausibly be linked to several aspects of the decision making process including stimulus encoding [28], accumulation of decision evidence [29], [30], the threshold level of evidence required for decision commitment [25], [31], [32], and/or the time devoted to response execution [cf. 16], [25]. However, no empirical studies to date have parsed how spontaneous fluctuations in arousal state or the attendant changes in neuromodulation affect such computationally-tractable aspects of accumulation-to-bound decision making.

Here, we examined how computational parameters of perceptual decision making are linked to slow, stimulus-independent fluctuations in pupil diameter. Pupil diameter provides a highly sensitive index of arousal state in conditions of constant luminance [33], and may indirectly reflect changes in the activity of subcortical neuromodulatory systems and their associated effects on neural processing [16], [23], [34], [35]. Indeed, much converging evidence indicates that ‘baseline’ pupil diameter correlates with specific behavioral metrics that are linked to tonic neuromodulator release, particularly with respect to the locus coeruleus-noradrenergic system [23], [36]–[40].

We measured pupil size while subjects performed a canonical perceptual decision making task involving motion discrimination, and decomposed observable behavior on this task into latent decision making parameters according to a prominent model of the decision process [41]–[45]. We found that changes in baseline pupil diameter were linked to between-trial variability in the rate of decision evidence accumulation with remarkable specificity: Periods of increased pupil diameter, indicative of heightened arousal, were characterised by greater variability in evidence accumulation rate, and did not relate to any other component of decision making. Pupil size also correlated with trial-by-trial task behavior in a manner specifically predicted of an index of variability in accumulation rate, and accounted for significant individual differences in this computational quantity. These findings provide a uniquely clear account of how arousal state impacts decision making, and may point to a strong relationship between tonic neuromodulation and behavioral variability.

## Results

**Figure 1:**
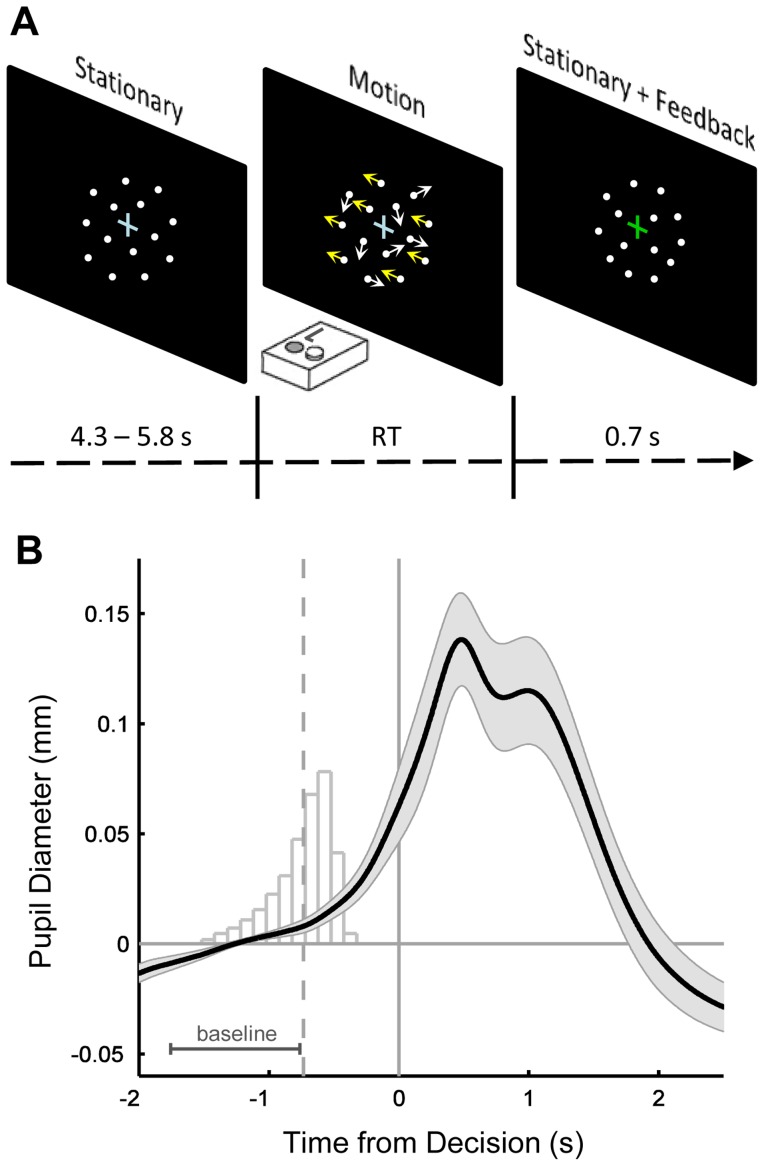
Random dot motion task. **A.** Schematic of a single trial of the random dot motion task. ‘Left’ or ‘right’ decisions were made with spatially compatible button presses under a deadline of 1500 ms after motion onset. Motion ceased upon response and was followed by an isoluminant mask of stationary dots, and feedback was indicated by a change in fixation cross color. **B.** Grand-average evoked pupil responses locked to the time of the decision. Histogram indicates the distribution of motion onset times relative to the subsequent response, after pooling trials across all subjects. Time period marked ‘baseline’ indicates the window over which baseline pupil diameter was calculated, in this case for trials characterized by the grand-mean response time (dashed vertical line). The minimum response-to-stimulus interval was 5 seconds; hence the prominent decision-related pupil dilation had sufficient time to return to baseline levels before the measurement period for baseline pupil diameter on the following trial. Shaded regions indicate S.E.M.

Twenty-six individuals performed a speeded response time (RT) version of a widely-used perceptual decision making paradigm, the random dot motion (RDM) task [46], [47]. The present version of the RDM involved two-alternative forced choice decisions about whether the dominant direction of motion of a cloud of moving dots was leftward or rightward (Figure 1A).

We sought to explore the relationship between pupil-linked arousal state and the computational foundations of the decision making process. Arousal is related to task engagement in a quadratic fashion [16]–[19], and where an individual resides on this ‘inverted-U’ curve is partly a function of task difficulty [48]. We thus attempted to limit individual differences in RDM discrimination difficulty, such that endogenous shifts in arousal had equivalent effects on task performance across our sample. To this end, subject-specific psychometric functions were estimated during an initial testing phase and the stimulus discriminability corresponding to 85% response accuracy was used for the remainder of the experiment (see *Materials and Methods*). During the main task phase, RTs on trials characterised by correct decisions (*M* = 723 ms, *SD* = 115 ms) were faster than RTs on incorrect trials (*M* = 807 ms, *SD* = 151 ms; paired-samples *t*-test, *t*_25_ = 6.78, *p*<0.0001).

### Pupil diameter is uniquely related to between-trial variability in the rate of evidence accumulation

In order to address our central question of how pupil-linked arousal state affects specific aspects of decision making, we focused on a baseline pupil diameter measure defined as the mean pupil diameter during the 1 s preceding motion onset on each trial. This measurement period is consistent with previous studies that have leveraged pupillometry to interrogate tonic arousal state [36]–[38], and yields a baseline measure that is distinct from the phasic, event-related pupil dilations that often follow salient task events (Figure 1B).

**Figure 2:**
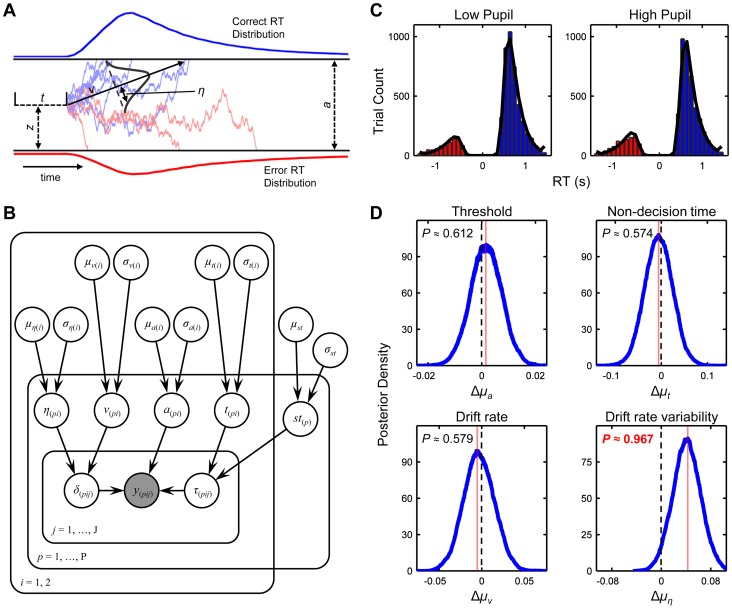
Drift diffusion modelling reveals a specific relationship between pupil diameter and variability in evidence accumulation rate. **A.** Schematic representation of the drift diffusion model (DDM). Noisy sensory evidence is accumulated over time from starting point *z* at mean drift rate *v*, until one of two thresholds (separated by *a*) is reached and a decision is made. Non-decision-related processing time is accounted for by *t*. Drift rate is normally distributed across trials with standard deviation *η*. Upper and lower panels show simulated RT distributions for correct and incorrect decisions. **B.** Graphical representation of the hierarchical DDM that was fit to the observed behavioral data. Nodes receiving arrow projections in the graph denote distributions that are parameterized by the parent nodes. Subscripts represent baseline pupil diameter bin *i*, participant *p*, and trial *j*. The shaded node *y* indicates the observed bivariate accuracy and response time data. *st* = between-trial variability in *t*. Starting point *z* was fixed at *a*/2 across *p*, *i* and *j* (not shown). **C.** Model predictions after hierarchical parameter estimation, illustrating the fit of the model to the observed data sorted into two bins according to baseline pupil diameter. Negative distributions indicate error RTs. Histograms illustrate observed data; overlaid lines show predictions. **D.** Bayesian posterior distributions representing the effect of pupil diameter bin (high – low) on selected model parameters. The *μ* notation refers to the estimated mean of the group-level distribution for each parameter. *P* denotes the mass of the effect distribution that is above or below zero. Vertical red lines indicate distribution modes.

We explored the relationship between this baseline pupil diameter measure and latent aspects of the decision process by fitting the drift diffusion model (DDM) to subjects' behavioral data. The DDM is a prominent mathematical model of simple two-choice decisions like those faced on the RDM task, and can parsimoniously account for full correct and error RT distributions across a diverse array of task settings [3], [41]–[44]. The model assumes that noisy sensory evidence is accumulated over time until one of two opposing boundaries is reached, at which point a decision is made in favor of the corresponding choice (Figure 2A). Core model parameters include the rate of evidence accumulation (drift rate) *v*, response boundary separation *a*, and non-decision time *t*. A central appeal of the DDM lies in the relatively clear mappings of these core parameters onto distinct psychological processes, such as the speed of information accumulation (*v*) and response caution (*a*). The full model also incorporates several sources of between-trial variability – for example, drift rate *v* is often assumed to vary across trials according to a normal distribution with standard deviation *η*. Variability parameters like *η* have proved necessary to explain specific aspects of the relationship between correct and error RT distributions [42], though little is known about what psychophysiological states might determine the magnitude of these parameters.

We fit the DDM to subjects' observed behavioral data via hierarchical Bayesian parameter estimation [49], [50]. This approach assumes that single-subject model parameters are randomly drawn from group-level parameter distributions, and deduces posterior probability densities for parameters at each hierarchical level by fitting to the entire group of subjects simultaneously (see *Materials and Methods*). Within this framework, the pooled group-level data effectively constrain parameter estimates for any one individual. In doing so, the hierarchical DDM confers a critical advantage over non-hierarchical approaches in that it yields more reliable parameter estimates when working with low trial numbers per subject/condition [49], [50]. In our case, trials for each subject were categorized according to whether they were preceded by high or low baseline pupil diameter, and the model was fit to RT and accuracy data from both resulting bins. In accordance with the exploratory nature of our research question, we let four key model parameters vary across pupil bin: *a*, *t*, *v* and *η* (Table 1; Figure 2B). The model fit the data from both pupil bins well (Figure 2C; Figure S1).

**Table 1:** Group-level hierarchical DDM parameter estimates.

	EAP		Posterior SD (×100)	
	Low Pupil	High Pupil	Low Pupil	High Pupil
***μ_a_***	0.108	0.109	0.414	0.412
***μ_t_***	0.526	0.520	2.152	2.100
***μ_v_***	0.204	0.201	1.311	1.306
***μ_η_***	0.123	0.164	1.662	1.652
***μ_st_***	0.077		0.851	

*EAP = expected* a posteriori.

*Posterior SD = standard deviation of marginal posterior*.

We tested for group-level effects of pupil diameter bin on the latent computations underying decision making behavior by interrogating posterior effect distributions that were estimated by the hierarchical model. These distributions were constructed to represent the pupil-related *change* in the group-level mean of each DDM parameter (see *Materials and Methods*). Analysis of the *a*, *t* and *v* effect distributions suggested that pupil diameter had no consistent relationship with any of these parameters: The probability mass of their respective distributions was roughly centred on zero (Figure 2D). By contrast, 96.7% of the mass of the *η* effect distribution lay above zero (Figure 2D), indicating that the *η* parameter tended to increase in magnitude with increasing pupil diameter. Thus periods of tonically increased pupil size, equivalent to a state of heightened arousal, were characterized by greater between-trial variability in the rate of evidence accumulation during perceptual decision making.

Notably, this group-level relationship between pupil diameter and *η* was relatively weak in strength; under standard frequentist statistical conventions, for example, a two-tailed hypothesis test would only yield a ‘significant result’ if more than 97.5% of the effect distribution was above zero. However, two considerations relating to the relative lack of constraint imposed by this particular hierarchical model should mitigate any concerns about the veracity of the effect. First, DDM parameters representing between-trial variability are difficult to reliably estimate at the single-subject level, particularly with the relatively low trial counts at our disposal. This issue can inflate the *uncertainty* associated with a parameter estimate, and lead to diminished estimates of effect reliability. Thus, some authors have tended to only estimate *η* at the group level [50], [51]. Accordingly, we found that the pupil/*η* effect was much more reliable when we took this approach (99.6% of the posterior effect distribution above zero; Figure S2). Critically, point estimates of the change in *η* across pupil bins (i.e. the means of the effect distributions) were similar for both models whereas the uncertainty associated with these estimates (i.e. the variance of each distribution) was markedly decreased in the group-only *η* model, thus suggesting that the additional uncertainty inherent in single-subject *η* estimation substantially diminished the estimated reliability of the reported effect.

A second point about the lack of constraint provided by our primary model is that the exploratory approach of letting 4 model parameters vary across pupil bins may have increased the probability that some meaningful *η*-related change in behavior was misattributed to any of the other three varying parameters, which would in turn dilute the estimated strength of any pupil/*η* effect. Indeed, examining the relationship in a more constrained manner by only letting *η* vary across pupil bin yielded a much more robust effect, with 99.8% of the estimated posterior effect distribution lying above zero (Figure S3).

The results from these alternative hierarchical modelling approaches should be viewed as complementary to our primary analysis, and convergent upon the conclusion that the *η* parameter of the DDM increases in magnitude with increasing baseline pupil diameter. In addition, a non-hiearchical version of the primary analysis that employed the same basic model constraints also corroborated the specificity of this relationship (Figure S4; see *Materials and Methods*).

A further method by which to verify the connection between pupil-linked arousal state and drift rate variability lies in interrogating the links between baseline pupil diameter and patterns of overt behavior that are thought to derive from a change in *η*. This approach, which we turn to next, is notably not affected by any concerns about model constraints or the framework for parameter estimation, and is not dependent on the specific bin sizes employed in our model-based analyses.

### Pupil-behavior relationships corroborate a link with variability in accumulation rate

Increased drift rate variability in the DDM has two cardinal effects on observable behavior. First, it leads to lower average response accuracy: A wider drift rate distribution with greater probability mass in its tails will produce a relatively higher proportion of trials with negative drift rate, and thus the probability across trials of the diffusion process terminating at the incorrect decision bound will be increased (assuming that the upper bound on the diffusion process represents the correct choice). Second, higher *η* specifically increases the discrepancy between average correct and error RTs, such that the latter are lengthened with respect to the former [42]. This selective widening of the gap between correct and error RTs again occurs because of the shift in probability mass to the tails of the drift rate distribution that comes with increased variability – at one extreme of the distribution, this leads to a relative increase in the number of very fast trials, very few of which are errors; at the other extreme, it leads to a relative increase in the number of very slow trials, a comparatively *large* proportion of which are errors.

**Figure 3:**
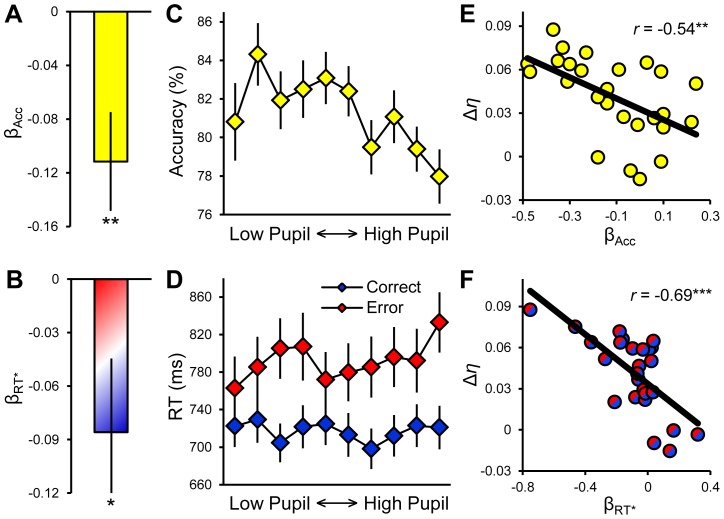
Pupil diameter correlates with response accuracy and the discrepancy between correct and error RTs. **A.** Mean of *β* coefficients from within-subjects logistic regressions of trial-by-trial response accuracy on baseline pupil diameter (Equation 1, *Materials & Methods*). **B.** Mean *β*s for the interaction term of within-subjects linear regression models designed to explore the relationship between baseline pupil diameter and RT (Equation 2, *Materials & Methods*). The effect illustrates the increased discrepancy between correct and error RTs as a function of increasing pupil diameter. **C,D.** Response accuracy (C) and RT (D) sorted within-subjects by baseline pupil diameter into 10 equal-sized bins, illustrating the predominantly linear nature of the relationships. **E.** The strength of the subject-specific relationship between pupil diameter and response accuracy (*β*_Acc_) was correlated with the change in drift rate variability from low to high pupil bins (Δ*η*). **F.** The strength of the relationship between pupil diameter and the discrepancy between correct and error RTs (*β*_RT*_) was also correlated with Δ*η*. Error bars = S.E.M. *** = *p*<0.001, ** = *p*<0.01, * = *p*<0.05.

We tested whether these behavioral trends – that together are diagnostic of an underlying change in drift rate variability – were observable in the trial-by-trial relationships between pupil diameter and task behavior in our data. Indeed, single-trial within-subjects logistic regressions of response accuracy on baseline pupil diameter (Equation 1, *Materials & Methods*) yielded *β* coefficients (*β*_Acc_) that were reliably negative (*t*_25_ = −2.90, *p* = 0.008; Figure 3A), thus establishing that accuracy decreased at higher levels of pupil diameter (Figure 3C). In addition, within-subjects linear regression models designed to test whether the nature of the relationship between baseline pupil diameter and RT depended on the accuracy of the current trial (Equation 2, *Materials & Methods*) yielded *β* coefficients for the relevant interaction term (*β*_RT*_) that were also reliably less than zero (*t*_25_ = −2.08, *p* = 0.048; Figure 3B). The direction of this result indicates, as expected, that the discrepancy between error and correct RTs grew as pupil size increased (Figure 3D). Collectively, these analyses highlight that pupil diameter was linked at the *trial-by-trial* level to accuracy and RT in ways that are specifically predicted of an index of between-trial variability in evidence accumulation rate.

We were also able to demonstrate via a series of additional control analyses that these pupil/behavior relationships could not be explained by any effects of previous-trial accuracy, eye gaze position or time-on-task on either behavior or baseline pupil diameter (Equations 3 & 4, *Materials & Methods*; Figure S5; Figure S6). The lack of influence of previous-trial accuracy is particularly noteworthy as it contrasts with the findings of prominent recent studies of the behavioral correlates of changes in baseline pupil diameter [37], [39], instead suggesting that the baseline variation in our study was more likely driven by slow, stimulus- and outcome-independent fluctuations in tonic arousal state.

As described in the previous section, the reliability of single-subject *η* parameter estimates should not be assumed *prima facie* when the DDM is fit to low trial-count data, as was the case in our study. The previously-calculated regression coefficients, which index the direction and strength of the relationships between pupil diameter and specific aspects of task behavior (*β*_Acc_, *β*_RT*_), provide an avenue by which to explore whether the per-subject *η* estimates do in fact account for pupil-linked variation in behavior in meaningful, expected ways. To do so, we correlated these regression coefficients, across subjects, with the degree of change in drift rate variability from low to high pupil bins (Δ*η*). As expected, accuracy *β* coefficients were negatively correlated with Δ*η* (*r* = −0.54, *p* = 0.005; Figure 3E) such that subjects whose accuracy decreased as a function of increasing pupil diameter also tended to show particularly large pupil-linked increases in *η*. Similarly, RT *β* coefficients were also negatively correlated with Δ*η* (*r* = −0.69, *p* = 0.0001; Figure 3F), indicating that subjects who showed a large pupil-linked increase in the discrepancy between correct/error RTs also tended to display large increases in *η* as pupil size increased. These between-subjects correlational results provide important evidence in support of the reliability of our single-subject *η* parameter estimates.

**Figure 4:**
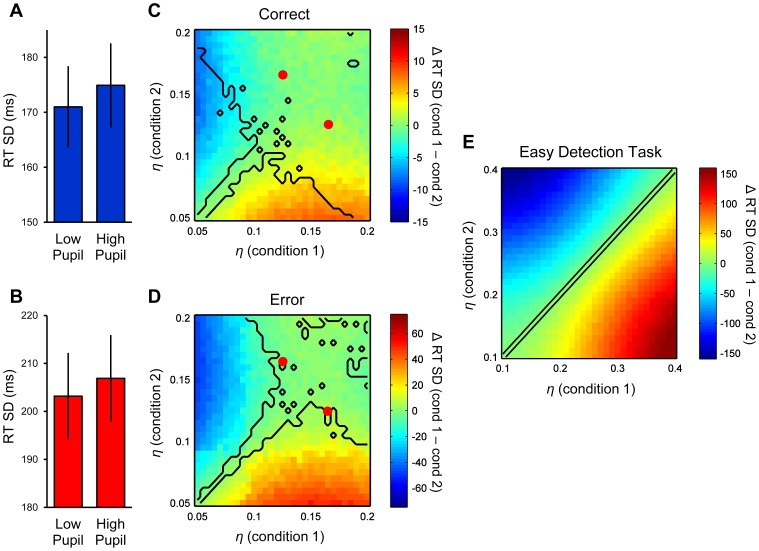
Simulated effects of between-trial variability in evidence accumulation rate on RT variability. **A,B.** Observed RT standard deviations in the low and high baseline pupil bins of our empirical study, for correct (A) and error (B) trials. Error bars = S.E.M. Although slight trends toward increased RT variability with increasing baseline pupil diameter existed for both RT-types, neither effect was statistically significant (both *p*>0.3). **C.** Heat map illustrating the effect of simulated changes in the *η* parameter of the DDM on correct-trial RT variability. Two conditions were constructed in which *η* was independently varied over a large range. Each pixel of the heat map represents the condition-related difference in the standard deviation of simulated correct RTs, averaged across 26 simulated subjects, for a specific pairwise comparison of *η* values (see *Materials & Methods*) – hotter colors indicate greater RT variability in condition 1 compared to condition 2. Black bounds enclose regions of the two-dimensional parameter space within which *η* was varied where the difference in RT variability was statistically significant at the group level (*p*<0.05, paired samples t-test, uncorrected). Red dots indicate the position in parameter space of the difference in *η* between the low and high baseline pupil bins of our empirical study, as estimated by the hierarchical DDM. **D.** Heat map illustrating the effect of simulated changes in the *η* parameter on error-trial RT variability. Method and conventions are the same as in *C*. **E.** Heat map illustrating the effect of simulated changes in *η* on RT variability during a simpler decision making task that is characterized by easy stimulus detection and an absence of errors [e.g. 36], [38]. Method and conventions are the same as in *C* and *D*, with the exception that behavior was simulated using a one-choice DDM with parameter sets devised from [51] (see *Materials and Methods*). All pixels in heat map show statistically significant condition-related differences, with the exception of those forming the identity line.

In contrast to previous reports employing easy stimulus detection tasks [36], [38], we did not observe a reliable relationship between baseline pupil diameter and RT variability (*p*>0.3 for paired-samples *t*-tests on both correct- and error-trial RT variability across high and low pupil bins; Figure 4A,B). Indeed, in line with this empirical finding coupled with our model fits identifying a robust link between pupil diameter and drift rate variability, subsequent DDM simulations indicated that increased *η* within a wide range of parameter values is also not reliably associated with a change in RT variability (Figure 4C,D; see *Materials and Methods*). How, then, can the present findings be reconciled with previous research [36], [38] that demonstrated a link between pupil size and RT variability during easy detection tasks? We employed a one-choice diffusion model [52] with high drift rate (thus approximating a simple decision making task in which stimulus detection is easy and errors do not occur) to simulate the overt behavioral consequences of increased accumulation rate variability in such settings (see *Materials and Methods*). Accordingly, increased *η* under these conditions was found to invariably correspond to increased RT variability (Figure 4E). Thus, the effect of a change in *η* on RT variability appears to be dependent on the nature of the current decision making context. Furthermore, these simulations reveal that previous findings [36], [38] that might initially be deemed incongruent with the present results are in fact consistent with the notion that pupil-linked arousal state reflects the level of variability in evidence accumulation rate.

### Pupil diameter indexes individual differences in the variability of evidence accumulation

**Figure 5:**
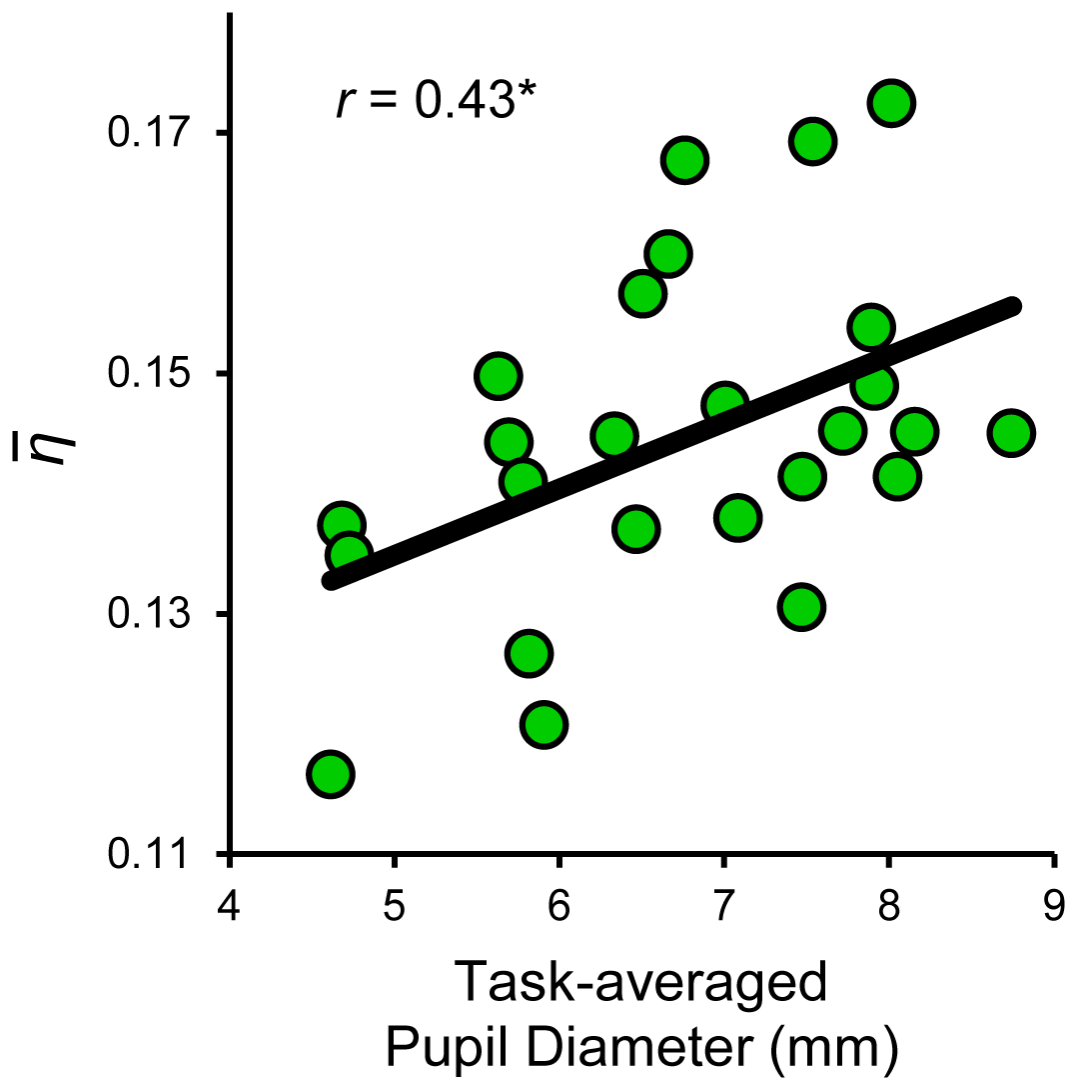
Pupil diameter explains individual differences in evidence accumulation rate variability. Baseline pupil diameter across the entire task correlated with mean drift rate variability across both pupil bins (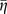). * = *p*<0.05.

Closer inspection of the pupillometric and model-based results revealed considerable variation between individuals in each of the considered measures. In a final analysis, we thus examined whether variation across subjects in the magnitude of drift rate variability during decision making could be partially explained by individual differences in average pupil size. Consistent with this intuition, baseline pupil diameter averaged over the entire task was positively correlated with mean drift rate variability across subjects (*r* = 0.43, *p* = 0.027; Figure 5). Notably, task-averaged pupil diameter did not correlate with any other DDM parameter (all *p*>0.1). Thus, in corroboration of the previously-reported within-subjects effects, individual differences in baseline pupil diameter were specifically linked to variability in the rate of evidence accumulation.

## Discussion

Arousal state has long been known to affect the manner in which organisms respond to stimulation from the environment [17]–[19], [22], and is closely regulated by the brain's neuromodulatory systems [16], [20], [21]. The strong associations between arousal, task-related behavior and neuromodulation have promoted speculation that arousal state might affect several discrete aspects of decision making involving the gradual accumulation of evidence over time [16], [25], [28], [31]. Here, we report several findings demonstrating that task-independent shifts in arousal state, as indexed by pupil diameter, are uniquely related to the amount of trial-by-trial variability in evidence accumulation rate during perceptual decision making: (i) periods of increased pupil diameter were characterised by greater variability in accumulation rate, as revealed by a prominent computational model of the decision process [41]–[44], [49]; (ii) pupil diameter correlated with specific behavioral signatures that collectively are diagnostic of a change in accumulate rate variability; and (iii) pupil diameter explained individual differences in this computational quantity. Our findings thus demonstrate that, far from exerting a variety of effects on separable aspects of decision making, changes in pupil-linked arousal state affect the decision process in a highly specific manner.

Trial-by-trial variability parameters were initially incorporated into sequential sampling models of decision making like the DDM because, without them, such models had difficulty accounting for commonly observed differences between correct and error response latencies [42]. However, in contrast to the four core parameters of the DDM (starting point, accumulation rate, response threshold, non-decision time), the psychophysiological determinants of changes in these variability parameters have not previously been identified. The present results are striking in that they afford the clearest indication to date that an established psychophysiological state determines the magnitude of between-trial variability in accumulation rate. Specifically, they imply that this model parameter reflects, at least in part, the impact of fluctuations in arousal state on the decision process, such that its magnitude increases in situations of tonically elevated arousal. This finding forges an explicit link between accumulation rate variability and a psychophysiological state that has long been known to determine task engagement [16]–[19], and thereby lays the foundation for an explanatory framework within which task-related or between-individual differences in this model parameter can now be interpreted. Furthermore, our results furnish new predictions regarding experimental manipulations that should specifically affect accumulation rate variability. We propose that manipulating tonic arousal (e.g. via presentation of white noise; [48]) during decision making could provide a fruitful line of future inquiry.

Although previous reports have highlighted a relationship between baseline pupil diameter and RT variability during easy stimulus detection tasks [36], [38], pupil diameter in the present study was solely correlated with response accuracy and the discrepancy between correct and error RTs. We were able to demonstrate that both sets of observations are in fact consistent with the proposal that pupil-linked arousal state determines the magnitude of variability in evidence accumulation rate during decision making. Diffusion model simulations revealed that the effect of a change in accumulation rate variability on observable RT variability is in fact dependent on the difficulty of the decision making task at hand. Under conditions of difficult stimulus discrimination when the diffusing process is sufficiently variable to frequently reach the incorrect response bound (as in the present study), the main consequence of an increase in *η* is a further decrease in response accuracy; however, the effect on RT variability in this context is not always reliable. By contrast, in a simpler decision making scenario characterized by high stimulus discriminability and an absence of errors, a primary consequence of higher *η* is a robust increase in RT variability. Thus we leveraged model simulations to establish changing accumulation rate variability as a plausible unifying mechanism that could mediate different, context-dependent relationships between pupil-linked arousal and overt decision making behavior.

The present results are also interesting in light of other recent pupillometric studies that relate stimulus- or outcome-evoked pupil dilation to latent aspects of the decision making process. For example, two recent publications [29], [32] suggest that the size of the stimulus-evoked dilation response during challenging decision making tasks scales positively with the amount of evidence required to commit to a decision, which is represented in the DDM by the response threshold parameter. Such findings contrast clearly with our identification of a link between ‘baseline’, pre-stimulus pupil size and variability in the rate of evidence accumulation. Considered together, this emerging literature lends renewed support to the long-held belief that changes in arousal affect behavior in different ways that are defined by the temporal scale over which they operate: slow tonic fluctuations in arousal appear to parameterize specific aspects of the neurocognitive state within which an upcoming decision will be made (present results; see also [37], [39]), whereas fast evoked changes in arousal appear to modulate the decision process *as it evolves* during a trial [29], [32]. This tonic/phasic distinction also has a clear neural basis in the activity patterns of various neuromodulatory nuclei [16], [21], [25], [26], [53].

Indeed, in light of potential links between pupil diameter and neuromodulatory tone [16], [23], [34], [35], our results identify tonic neuromodulation as a plausible neurophysiological substrate for changes in accumulation rate variability during decision making. Several sources of evidence have recently implied a strong link between catecholaminergic neuromodulation and across-trial variability in single-cell neuronal activity [54], [55]. Our findings complement these reports by highlighting what may be a higher-level manifestation of neuromodulatory effects on processing variability, and in doing so reinforce the notion that neuromodulator availability in cortex is likely an important determinant of the precision with which sensory information is encoded and leveraged to guide behavior.

What specific biophysical mechanism might mediate a pupil-linked increase in accumulation rate variability? One candidate is the effect of neuromodulators on the gain of neural activity [16], [23]–[25]. Neural gain acts to amplify neural communication, such that excited neurons become even more active and inhibited neurons become even less active under high gain states. Transient increases in gain can be highly advantageous for behavior when applied at the right time [16], [27], but high gain can also propagate the influence of noise across all levels of processing when applied indiscriminately [16], [25]. Tonically high neuromodulator release, likely marked by periods of large pupil diameter in our study, is equivalent to this type of indiscriminate increase in global neural gain [23]. The heightened influence of noise that arises from tonically high gain may naturally load onto between-trial variability in accumulation rate in typical fits of the DDM to behavioral data, which coheres well with the relationship we observed between this parameter and pupil diameter.

The DDM also contains a parameter *s* that represents *within*-trial variability in accumulation rate, and is another plausible candidate parameter that might be affected by tonic changes in neural gain. We did not directly examine whether this was the case in our data because, in fits of the DDM to observed behavior, *s* acts to scale all other model parameters across conditions and subjects and hence is fixed at an arbitrary value [45]. Nonetheless, our simulations indicate that increased *s* would result in *faster* error compared to correct response times and hence would not be sufficient to explain our observed relationships between pupil diameter and behavior (Figure S7). Thus, two possibilities remain that we do not currently arbitrate between. First, elevated arousal and neural gain may increase between- *and* within-trial variability in accumulation rate, and both effects interact to produce the specific behavioral signatures that we observed at high levels of pupil diameter. Alternatively, arousal state may be uniquely related to the between-trial variability parameter. Although the former prospect seems more intuitively likely given the global, indiscriminate nature of tonically increased neural gain, the latter has some precedent in classic psychological arousal theory. Specifically, high arousal has been thought to invoke highly labile attentional allocation at a global level, but coupled with highly fixed attention at a local level [17], [18]. In the context of the present study, this global/local distinction could manifest in a more variable decision process *between* trials, but without any additional variability in processing *within* individual trials.

Lastly, although the primary consequence of increased accumulation rate variability in our decision making context (i.e. decreased response accuracy) is clearly maladaptive, heightened processing variability likely holds certain adaptive advantages in other settings. For example, in situations where the value of each choice option fluctuates unpredictably, heightened neural noise can drive exploration of the full range of alternatives and lead to a revised, more accurate internal model of the environment [56]. Indeed, arousal state and neuromodulators are thought to play a key role in mediating shifts in this so-called exploration/exploitation trade-off [16], [37], [56]. Our findings suggest that adjustments to evidence accumulation rate variability may reflect a mechanism, at the level of the decision process, by which such a trade-off is regulated.

## Materials and Methods

### Ethics statement

The study was approved by the ethics committee of the Leiden University Cognitive Psychology department, and all protocols were conducted in accordance with the Declaration of Helsinki. All subjects provided written informed consent prior to taking part.

### Subjects

Twenty-six individuals [age range: 18–29; mean age: 22.5± SD of 2.7 years; 22 female] participated in the study. All subjects had normal or corrected-to-normal vision, no history of psychiatric illness, and spoke fluent English. A further 7 individuals were recruited but not fully tested after failing to reach an initial criterial level of task performance (see below).

### Behavioral protocol

We employed a speeded RT version of the random dot motion (RDM) paradigm. During task performance, subjects were instructed to maintain fixation at all times on a centrally presented light-blue cross (occupying 0.36° of the visual angle) and decide whether the dominant direction of motion of a cloud of moving dots was leftward or rightward. The directions were equiprobable and randomly selected across trials. The dots were white with a size of 3×3 pixels, moved within a circle of 5° diameter at a speed of 5°/s and a density of 16.7 dots/degree^2^/s, and were presented on a black background [6]. For the first three frames of a given trial, all dots were located in random positions. For each of these frames the dots were repositioned after two subsequent frames (dots in frame 1 were repositioned in frame 4, those in frame 2 were repositioned in frame 5, etc.). For each dot, the new location could be either random or determined by the direction of motion on that trial. The probability with which each dot moved in this pre-determined direction is defined as coherence: At a coherence of 50%, each dot has a 50% probability of participating in the motion signal, every third frame.

All stimuli were presented via the Psychophysics Toolbox Version 3.0.8 [57], [58] for Matlab. A 47 cm-wide LCD monitor was employed that operated at 60 Hz and a resolution of 1680×1050 pixels. Subjects were seated 60 cm from the monitor with their heads supported by a chinrest to ensure constant viewing distance and position throughout task performance. Testing was conducted under moderate levels of ambient light (∼18 cd/m^2^).

Subjects were instructed to perform the task ‘as quickly and as accurately as possible’, and indicated their response on a given trial by pressing one of two spatially compatible keyboard keys (‘Ctrl’ right or left) with their right or left index finger. Upon response execution, the moving dots were replaced with an isoluminant mask of stationary dots that were randomly distributed within the aperture of the 5° circle and displayed until the start of the following trial. In addition, the colour of the fixation cross changed for 700 ms post-response according to the accuracy of that response: green if correct, light-red if incorrect. All fixation cross colours were selected from the Teufel colours [59] in order to approximate isoluminance throughout motion discrimination, feedback and inter-stimulus interval. Lexical feedback was provided in plain red font when responses were quicker than 100 ms post-stimulus onset or when a response had not been made after 1,500 ms (“too fast” and “too slow”, respectively), and these trials were not included in any analysis. The response-to-stimulus interval (RSI), including feedback duration, was drawn from a uniform distribution with a range of 5 to 6.5 s – pilot testing revealed that this extended RSI was sufficient to allow evoked pupil responses to return to baseline levels before measurement of baseline pupil diameter on the subsequent trial (see Figure 1B). The RSI distribution during initial practice blocks was shortened to respective bounds of 1.6 s and 3.1 s in order to minimize the total length of the testing session.

After receiving initial automated task instructions, subjects completed a practice block of 40 trials at 50% dot coherence. If an accuracy level of 70% was not achieved, they were required to repeat the practice block until this threshold level of task performance was attained. In such circumstances, a verbal instruction was given to slow down and allow more time to process the stimulus. Subjects who failed to achieve the 70% accuracy cut-off after 3 practice blocks were not tested further and were excluded from the study (*n* = 3). Even if the criterion level of accuracy was reached, subjects were afforded the opportunity to perform another practice block if they wished. An average of 1.6±0.8 practice blocks were performed by subjects who progressed to the next phase of testing.

In order to match the difficulty of the RDM discrimination across subjects, the practice blocks were followed by a block of 200 trials of randomly interleaved dot coherences (0, 10, 20, 40 or 80% coherence, 40 trials each). We fit the proportional-rate diffusion model to the mean RT and accuracy data yielded therein using a maximum likelihood procedure [6], [60]. The proportional-rate model is a simple, low-parameter version of the diffusion model that can be used to quickly estimate individual subjects' psychometric and chronometric functions [60]. For each participant, the dot coherence corresponding to 85% accuracy was interpolated from their model-estimated psychometric curve and used in all remaining experimental blocks. Mean accuracy across remaining trials was marginally but significantly lower than this target level (*M* = 81.9, *SD* = 5.5%; one-sample *t*-test, *t*_25_ = −2.83, *p* = 0.009), likely due to slight misestimation of the true psychometric functions. Nonetheless, this procedure appeared to fulfil its function of limiting between-subjects variance in experienced task difficulty. Notably, subject-specific coherence level did not correlate significantly with mean accuracy, drift rate variability or baseline pupil diameter across individuals (all *p*>0.1), suggesting that this difficulty calibration process was not a primary determinant of any residual between-subjects variance in task performance and/or arousal state. The performance of 4 subjects on this preliminary block was too poor for estimation of a reliable psychometric function (mean estimated dot coherence for 85% accuracy: 85.3±17.5%). These subjects were not tested further and were excluded from all analyses.

All remaining subjects (*n* = 26) were then administered 500 trials of the RDM task, broken into 5 blocks of 100 trials. Each block began with presentation of the fixation cross and a stationary mask of dots for 10 s, allowing pupil size to stabilise before onset of the first trial. Mean block duration was 10.9±0.2 minutes. Subjects were allowed short rest periods between blocks, at the end of which they were reminded to maintain fixation during the following block and perform the task as quickly and accurately as possible. Total duration of the testing session, including practice and psychometric function phases, was approximately 2 hours and 15 minutes.

### Pupillometry

Pupil diameter and gaze position were recorded during all non-practice blocks, at a sampling rate of 250 Hz. Gaze position was calibrated via the standard Eyelink procedure before the start of each block of trials. Pupil diameter was originally recorded in arbitrary pixels. We derived a subject-specific scaling factor by measuring the size of a ‘model pupil’ of precisely known diameter, under the same physical conditions (camera focus, distance to camera) used for testing that subject. This scaling factor could then be used to convert the pupillometric time series to units of mm. Eye-blinks and other noise transients were removed offline using a custom linear interpolation algorithm that restricted interpolation to periods of consecutive data loss that were shorter than 1 second. We then identified remaining artifactual samples by applying amplitude (any sample <1 mm), gradient (any difference in consecutive samples >0.02 mm) and gaze position (any sample in which gaze deviated from fixation by >5°) thresholds to the interpolated data. An average of 1.6±3.0% of trials contained at least one artifactual sample within the window −1 to 0 s relative to dot motion onset and these were excluded from all analyses. Baseline pupil diameter was defined on each remaining trial as the mean pupil diameter during the 1 s preceding motion onset [36], [38].

### Drift diffusion modelling

We decomposed behavioral data from the RDM task into latent parameters of the decision process via the drift diffusion model (DDM; [41]–[43], [45], [49]). The DDM assumes that for two-alternative forced choice decisions, noisy sensory evidence is accumulated from a starting point *z* at drift rate *v*. The moment-to-moment noise during accumulation is governed by the *s* parameter, which refers to the standard deviation of a zero-mean Gaussian distribution from which random increments to the deterministic component of the accumulation process (represented by *v*) are drawn. The *s* parameter acts to scale all other parameters in the model across conditions and individuals. Hence it tends to be fixed in the vast majority of DDM fits at the arbitrary value of 0.1, and this was the case in our study. The DDM assumes that a response is initiated when a criterial amount of evidence has been accumulated to pass one of two opposing boundaries corresponding to either choice option. The distance between these boundaries is referred to as the response threshold *a*. The model ascribes all non-decision-related processing such as sensory encoding and response execution to a non-decision time parameter *t*. Furthermore, the full DDM identifies three different sources of trial-to-trial variability in order to account for the full range of empirically-observed RT distributions: variability in drift rate (*η*), variability in starting point (*sz*), and variability in non-decision time (*st*).

We fit the DDM according to a hierarchical Bayesian application of the model [49], which estimates single-subject DDM parameters and group-level distributional (mean, variance) parameters simultaneously via Markov chain Monte Carlo (MCMC) sampling techniques. Within this framework, group-level data effectively constrain parameter estimates for any one individual. In doing so, the hierarchical DDM confers a critical advantage over non-hierarchical approaches in that it yields more reliable parameter estimates when working with low trial numbers [49], [50]. This advantage is especially decisive when a between-trial variability parameter is of interest, as emerged to be the case here, since reliable estimation of such parameters using non-hierarchical model fitting typically requires a particularly large number of observations.

For each subject, trials were collapsed across dot motion direction and classified by response accuracy (correct, error) before model fitting. Preliminary analyses indicated that relationships between pupil size and behavior were predominantly linear (Figure 3C,D). Trials were therefore sorted by baseline pupil diameter and pooled into *two* bins containing the lowest 2/5ths and highest 2/5ths of diameter values for each subject (referred to as ‘low’ and ‘high’ pupil bins in the main text). These bin bounds produced a reasonable trade-off between maximising trial counts for modelling (average of 160±13 correct and 36±11 error trials per bin after artifact rejection) and yielding a robust effect of pupil bin on overt behavior.

The hierarchical DDM, implemented via the Metropolis-Hastings MCMC sampling algorithm in WinBUGS [61], was fit to RT and accuracy data from both pupil bins, pooled across all participants. Six independent chains of 15,000 iterations each were generated from the full posterior distribution, with the first 10,000 iterations of each chain discarded as burn-in. Chain convergence was assessed by computing, for each parameter, the 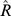 criterion comparing between- to within-chain variance [62]. Initial fitting attempts indicated that the *sz* parameter failed to converge across simulation chains; hence this parameter was omitted from the model [50]. 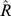 statistics for all remaining parameters were lower than 1.05, indicating good convergence. Model fit was visualized by generating posterior predictive data from the full posterior distribution of the parameters; specifically, we simulated 1,000 new datasets on the basis of 1,000 samples from the posterior, pooled the simulations across all 1,000 datasets, and plotted the pooled simulations against the observed data (Figure 2B; Figure S2).

We tested for the presence of baseline pupil bin effects on parameters representing the group-level means of the *a*, *t*, *v* and *η* parameters by computing posterior effect distributions of the change in each parameter from low to high pupil bins. That is, a difference measure (high minus low bin) for each group-level mean was derived from each of the 30,000 samples from the full posterior, and the effect distributions were constructed from these difference scores. Effect size was quantified for each effect distribution by computing the probability mass that lay above or below zero – if most of the mass was above zero then that parameter likely increased with increasing pupil diameter, whereas the parameter likely decreased with increasing pupil diameter if most of the mass was below zero.

Three other versions of the DDM were also fit to the data in order to further verify the primary results. First, we imposed an additional constraint on the above hierarchical DDM such that *η* was only estimated at the group level, thereby facilitating more stable estimation of the group-level distributions for this parameter [cf. 50] (Figure S2). Second, we imposed the restriction that only *η* was free to vary across pupil bin, thereby interrogating the relationship between *η* and baseline pupil diameter in a more constrained manner (Figure S3). Third, we fit the full non-hierarchical DDM to the behavioral data using the DMAT toolbox [63] (Figure S4). Specifically, we fit the non-hierarchical model to each subject's behavioral data by computing five RT quantiles (0.1, 0.3, 0.5, 0.7, 0.9) for both correct and error responses within each pupil bin, computing their likelihood given model predictions, and optimizing model fit via the standard DMAT Simplex minimization routine [63]. As was the case with our primary hierarchical analysis, the *a*, *v*, *t* and *η* parameters were free to vary across pupil bins while all other parameters were fixed across pupil bins.

### Model simulations

The overt behavioral consequences of incremental changes to specific parameters of the decision process were explored by using the DDM to generate simulated accuracy and RT data. We randomly drew sets of DDM parameters for 26 mock ‘subjects’ from the estimated group-level parameter distributions (without marginalizing over each pupil bin) of our previously-fit hierarchical DDM. Employing the same sample size as our empirical study and drawing parameter sets from the fitted HDDM effectively ensured that any information gleaned by simulation could be used to make inferences about how changes to particular DDM parameters would affect overt behavior within our specific empirical setting. Next, we sought to address two questions by adjusting specific model parameters of interest and simulating behavioral data. First, does a change in the *η* parameter of the DDM effect a change in observed RT variability? To address this question, we constructed two conditions within which *η* was systematically varied over a large range (from 0.05 to 0.2 in 0.005 increments), in unison for the entire sample of simulated subjects, while holding all other parameters constant. We varied *η* independently within each condition in such a way as to cover the entire two-dimensional space of pairwise comparisons within the broad range of *η* values, and compared the variability in both correct and error RTs between the two conditions (Figure 4). The second question centred on whether the *s* parameter (representing *within*-trial variability in evidence accumulation) is capable of producing patterns of behavior that are consistent with a change in *η* (i.e. changes in accuracy and the gap between correct and error RTs; see main manuscript). Again, we constructed two conditions: one ‘reference’ condition within which *η* and *s* were fixed (at 0.125 and 0.1, respectively, thus mimicking the ‘low pupil’ condition of our empirical study), and a second in which both *η* and *s* were systematically varied over moderate ranges (0.06–0.19 for the former, 0.07–0.13 for the latter, both in increments of 0.005). As with the *η*/RT variability analysis, *η* and *s* were varied independently to cover the entire two-dimensional space of pairwise comparisons within these ranges; here, however, we compared response accuracy and the difference between mean error and correct RTs between the ‘varying’ condition and the fixed reference condition (Figure S6). In both of the above analyses, 50,000 trials were simulated per condition per subject; this large number circumvented any noise introduced by low trial numbers and yielded reasonable estimates of the true nature of the underlying effects of interest.

In an effort to reconcile the results reported here with previous research demonstrating a link between pupil size and RT variability on easy detection tasks [36], [38], we also explored the effects of a change in *η* on RT variability in a simpler decision making context. To this end, we simulated further sets of behavioral data in the manner outlined above, but now using a one-choice diffusion model under conditions of high stimulus discriminability (thus simulating an easy decision making context in which RTs are typically fast and error responses absent; [36], [38]). The standard two-bound diffusion model could also be employed to simulate such situations, and would yield very similar results under conditions of sufficiently high drift rate. Parameter sets were again devised for 26 mock subjects; this time, they were randomly drawn from normal distributions with group means and across-subject variances that were based on a one-choice diffusion model fit to behavior during an easy visual detection task, as reported in a previous publication [52] (mean ± *SD*: *v* = 0.777±0.191; *a* = 0.136±0.017; *t* = 0.253±0.034; *st* = 0.136±0.037). Again, two conditions were constructed within which *η* was systematically varied over a large range (from 0.1 to 0.4 in 0.01 increments), and condition-related differences in RT variability were examined (Figure 4E). This range of *η* extends well beyond that encompassed by the distribution of fitted *η* values in [52] (mean ± *SD*: 0.262±0.076), and thus likely encompasses the full range of realistic *η* values in this particular decision making context.

### Pupillometric analyses

We interrogated trial-by-trial links between baseline pupil diameter and overt task behavior via within-subjects regression analyses. The relationship between pupil diameter and response accuracy was probed via logistic regressions of single-trial accuracy on pupil diameter, according to the following equation:
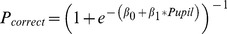
(1)where *P*_correct_ is the probability of making a correct motion discrimination decision, *Pupil* is z-scored single-trial baseline pupil diameter, and for all equations, *β*_i_ are fitted regression coefficients. This approach yielded per-subject regression coefficients representing the strength and direction of the relationship between pupil diameter and response accuracy (β_1_ in Equation 1; referred to as *β*_Acc_ in main text). To examine the single-trial relationship between baseline pupil diameter and RTs, we fit the following linear regression model:
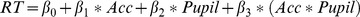
(2)where *RT* indicates z-scored, log-transformed single-trial RT, *Acc* is a binary indicator variable representing single-trial accuracy (coded as 1 = correct, 0 = error), and the final term represents the interaction between the *Acc* and *Pupil* predictors. For the present purposes, the most interesting quantity from this model is the regression coefficient estimated for the interaction term (*β*_3_ in Equation 2; referred to as *β*_RT*_ in main text), as it indicates whether the nature of the relationship between pupil size and RT changes as a function of the accuracy of the current trial – or alternatively, whether any discrepancy between correct and error RTs changes as a function of baseline pupil diameter. Lastly, we also fit two expanded regression models, according to the following equations:
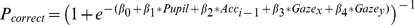
(3)

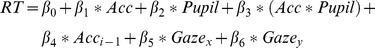
(4)where *Acc_i-1_* is a binary vector representing previous-trial accuracy, *Gaze_x_* is z-scored mean pre-stimulus gaze position along the horizontal axis, and *Gaze_y_* is z-scored mean gaze position along the vertical axis. The additional terms in these models make it possible to examine the relationships between baseline pupil diameter and accuracy (Equation 3) and RT (Equation 4) while statistically controlling for any sequential behavioral/pupillometric effects determined by previous-trial accuracy, and also for any effects of baseline-period gaze position on pupil diameter or behavior (see Figure S5). Group-level effects on fitted *β* values for individual predictors were tested via one-sample *t*-tests (*H_0_*: *β* = 0).

Uni-linear between-subjects correlations were conducted using Pearson's *r*. Average baseline pupil diameter for a given subject was defined as the mean of all single-trial baseline pupil measures for that subject pooled across the entire task, thus yielding a per-subject estimate of pupil size that could be subjected to individual differences analysis. Somewhat surprisingly, this average pupil metric did not significantly correlate with subject age (*r* = −0.28, *p* = 0.16), perhaps because the age range of our sample was quite narrow. Subject-specific average DDM parameters were calculated by taking the mean of the relevant marginal posterior for each subject, without marginalizing over pupil bin. The change in drift rate variability from low to high pupil bins (Δ*η*) was calculated by constructing subject-specific posterior effect distributions in the manner outlined for group-level effects above, and extracting the mean of each distribution.

## Supporting Information

Figure S1 **Posterior predictive data from the primary hierarchical model illustrating model fit for each single subject.** Negative distributions indicate error RTs. Histograms illustrate observed data; overlaid lines illustrate predicted data. Text at inset indicates subject number and estimated pupil-linked change in drift rate variability (Δ*η*) for that subject.
(TIF)

Figure S2 **Graphical representation and posterior effect distributions from an alternative hierarchical model in which drift rate variability was only estimated at the group level.** ***A*** **.** Directed acyclical graph with the same conventions as Figure 2B. Note that the *η* parameter representing between-trial variability in drift rate is located outside the participant loop, and thus only estimated at the group level. ***B*** **.** Posterior distributions representing the effect of pupil diameter bin (high – low) on selected parameters from the alternative model depicted in (A). As in Figure 2D of the main manuscript, the *μ* notation refers to the estimated mean of the group-level distribution for each parameter while *P* denotes the mass of the effect distribution that is above or below zero. Vertical red lines indicate the mode of each distribution.
(TIF)

Figure S3 **Graphical representation and effect distribution from a second alternative hierarchical model in which only drift rate variability was free to vary across pupil bins.** ***A*** **.** Directed acyclical graph with the same conventions as Figure 2B and Figure S2A. Note that the *η* parameter is located inside the pupil bin loop, but all other parameters are outside this loop. ***B*** **.** Posterior distribution from the alternative model depicted in (A), representing the effect of pupil diameter bin (high – low) on *η*. The *μ* notation refers to the estimated mean of the group-level distribution for each parameter while *P* denotes the mass of the effect distribution that is above or below zero. Vertical red lines indicate the mode of the distribution.
(TIF)

Figure S4 **Parameter estimates from a fit of the full non-hierarchical drift diffusion model to each subject's observed data.** Basic model constraints mimicked those of the hierarchical diffusion model depicted in Figure 2 of the main manuscript: the model was fit to data from both high and low baseline pupil bins, and *a*, *t*, *v*, and *η* were free to vary across pupil bin. See *Materials & Methods* for details of the fitting procedure. Error bars = S.E.M. * = *p*<0.05.
(TIF)

Figure S5 **Previous-trial accuracy and gaze position do not account for the observed relationships between pupil diameter and overt behavior.** **A.** Grand-average evoked pupil responses locked to the time of the decision, for both correct and error trials. Dashed vertical lines indicate the grand-mean RTs for both response types, shaded regions indicate S.E.M. Note the error-related increase in pupil diameter, relative to correct trials, is sustained well beyond the latency of peak dilation. **B.** This sustained error-evoked increase in pupil diameter manifested as larger baseline pupil diameter values, on average, when the previous trial was an error compared to a correct decision. **C.** Mean *β* coefficients from an expanded logistic regression model quantifying the relationship between single-trial response accuracy and a selection of predictors (Equation 3 in main text). Notably, neither previous-trial accuracy nor pre-stimulus gaze position predicted current-trial accuracy, though the relationship between baseline pupil diameter and accuracy remained present. **D.** Mean *β* coefficients from an expanded linear regression model with single-trial RT as the dependent variable, including the same additional covariates as in (C) (Equation 4 in main text). The previously-observed pupil * current-trial accuracy interaction effect remained intact in the presence of the additional terms. The highly significant effects for the *Accuracy (t)* and *Accuracy (t-1)* predictors indicate that RTs were slower on error compared to correct trials (see also main text), and that RTs slowed down after errors, respectively. Error bars = S.E.M. *** = *p*<0.001. * = *p*<0.05 (one-tailed).
(TIF)

Figure S6 **Effects of time-on-task on task behavior and pupil diameter.** **A,B,C.** Plots depicting the average response accuracy (A), correct and error RTs (B) and baseline pupil diameter (C) for each block of task performance. Analysis of the linear effect of task block on each measure revealed no significant effects (all *p*>0.1). Error bars = S.E.M.
(TIF)

Figure S7 **Dissociating the effects of between- and within-trial variability in evidence accumulation rate on observable behavior.** **A**. Heat map illustrating the effect of simulated changes in the *η* and *s* parameters of the drift diffusion model on response accuracy. Two conditions were constructed: one ‘reference’ condition in which *η* and *s* were fixed (at 0.125 and 0.1, respectively, thus mimicking the ‘low pupil’ condition of our empirical study), and a second in which both *η* and *s* were systematically varied over moderate ranges. Each pixel of the heat map represents the condition-related difference in response accuracy, averaged across 26 simulated subjects, for a specific pairwise comparison of one pair of *η* and *s* values with the fixed reference condition (see *Materials and Methods*) – hotter colors indicate higher response accuracy in the ‘varying’ condition compared to the reference condition. The black cross indicates the position of the reference condition in the two-dimensional parameter space within which *η* and *s* were varied. The plot indicates that an increase in either, or both, of the variability parameters leads to decreased response accuracy. ***B***. Heat map illustrating the effect of simulated changes in the *η* and *s* parameters on the difference between mean error and correct RTs. Method and conventions are the same as in (A); hotter colors indicate comparatively slower error compared to correct RTs in the ‘varying’ compared to the reference condition. The plot indicates that the *η* and *s* parameters have opposite effects on the error/correct RT discrepancy: increased *η* produces slower error compared to correct RTs, while increased *s* produces *faster* error compared to correct RTs.
(TIF)
