# Supplementary figures and images for: Pupil-Linked Arousal Determines Variability in Perceptual Decision Making
Source: PLoS Comput Biol. 2014 Sep 18;10(9):e1003854. doi: 10.1371/journal.pcbi.1003854 (PMC4168983; doi:10.1371/journal.pcbi.1003854)

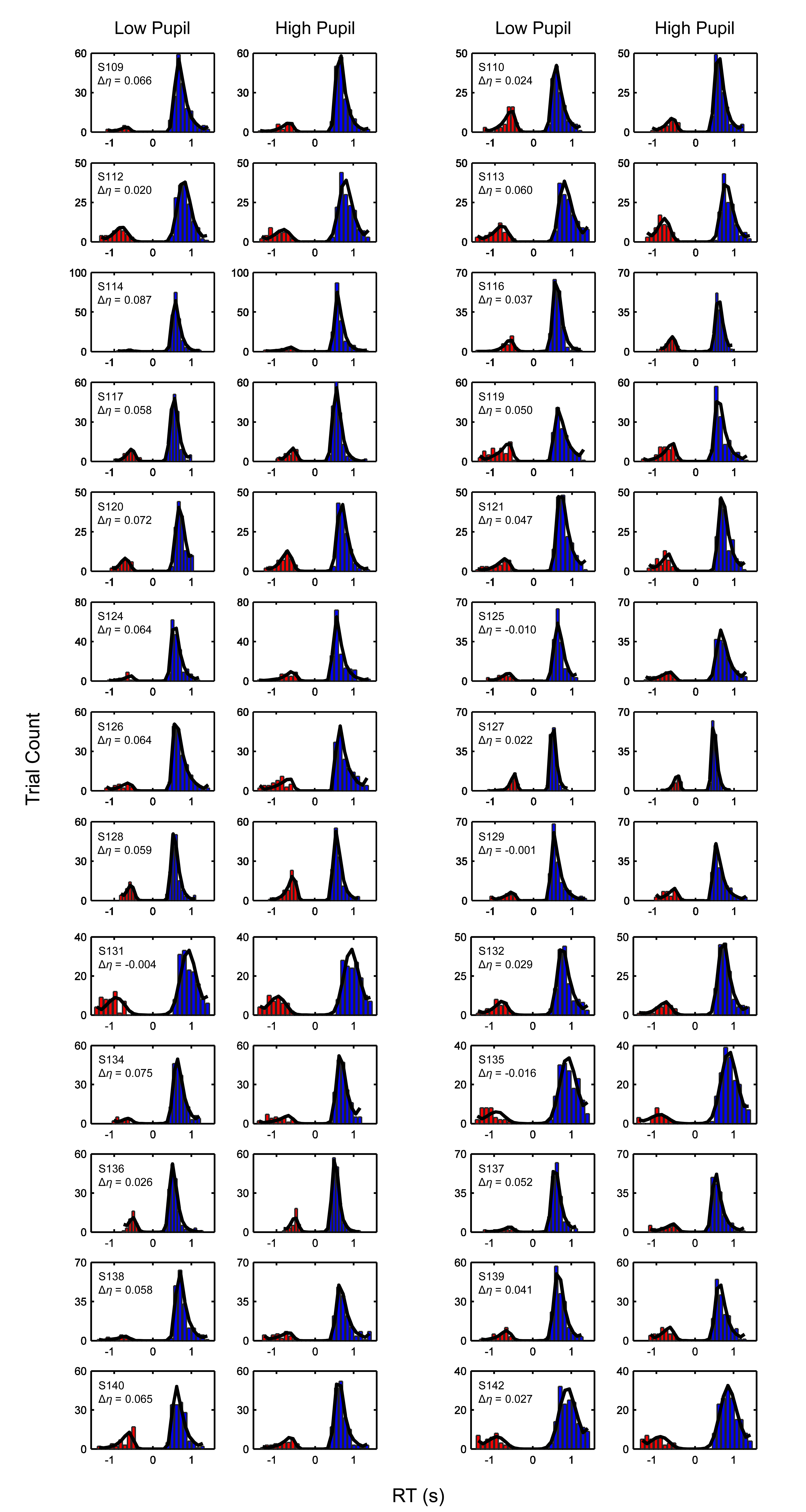

Supplement: Figure S1 — Posterior predictive data from the primary hierarchical model illustrating model fit for each single subject. Negative distributions indicate error RTs. Histograms illustrate observed data; overlaid lines illustrate predicted data. Text at inset indicates subject number and estimated pupil-linked change in drift rate variability (Δη) for that subject. (TIF) [file pcbi.1003854.s001.tif]

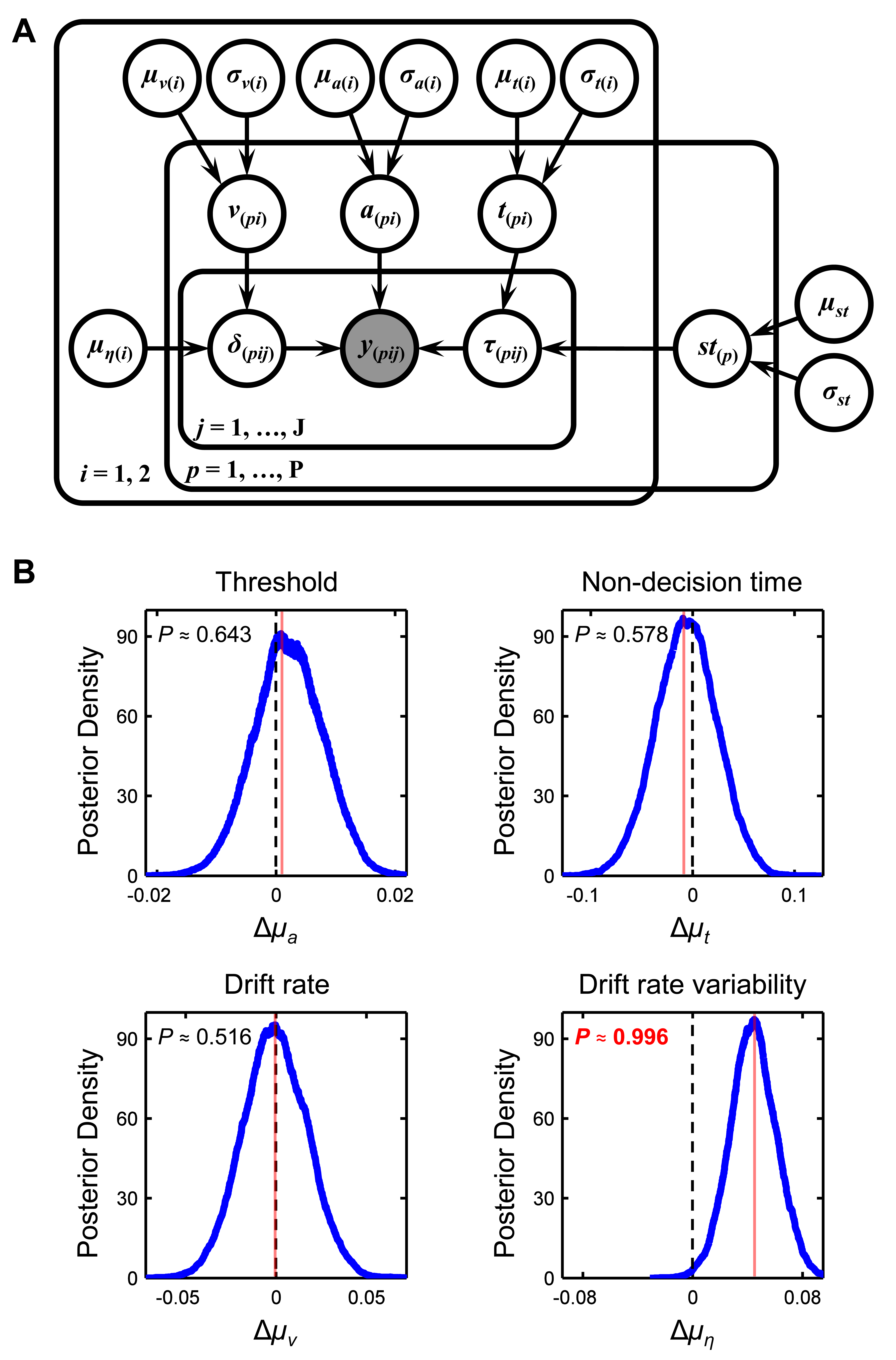

Supplement: Figure S2 — Graphical representation and posterior effect distributions from an alternative hierarchical model in which drift rate variability was only estimated at the group level. A . Directed acyclical graph with the same conventions as Figure 2B. Note that the η parameter representing between-trial variability in drift rate is located outside the participant loop, and thus only estimated at the group level. B . Posterior distributions representing the effect of pupil diameter bin (high – low) on selected parameters from the alternative model depicted in (A). As in Figure 2D of the main manuscript, the μ notation refers to the estimated mean of the group-level distribution for each parameter while P denotes the mass of the effect distribution that is above or below zero. Vertical red lines indicate the mode of each distribution. (TIF) [file pcbi.1003854.s002.tif]

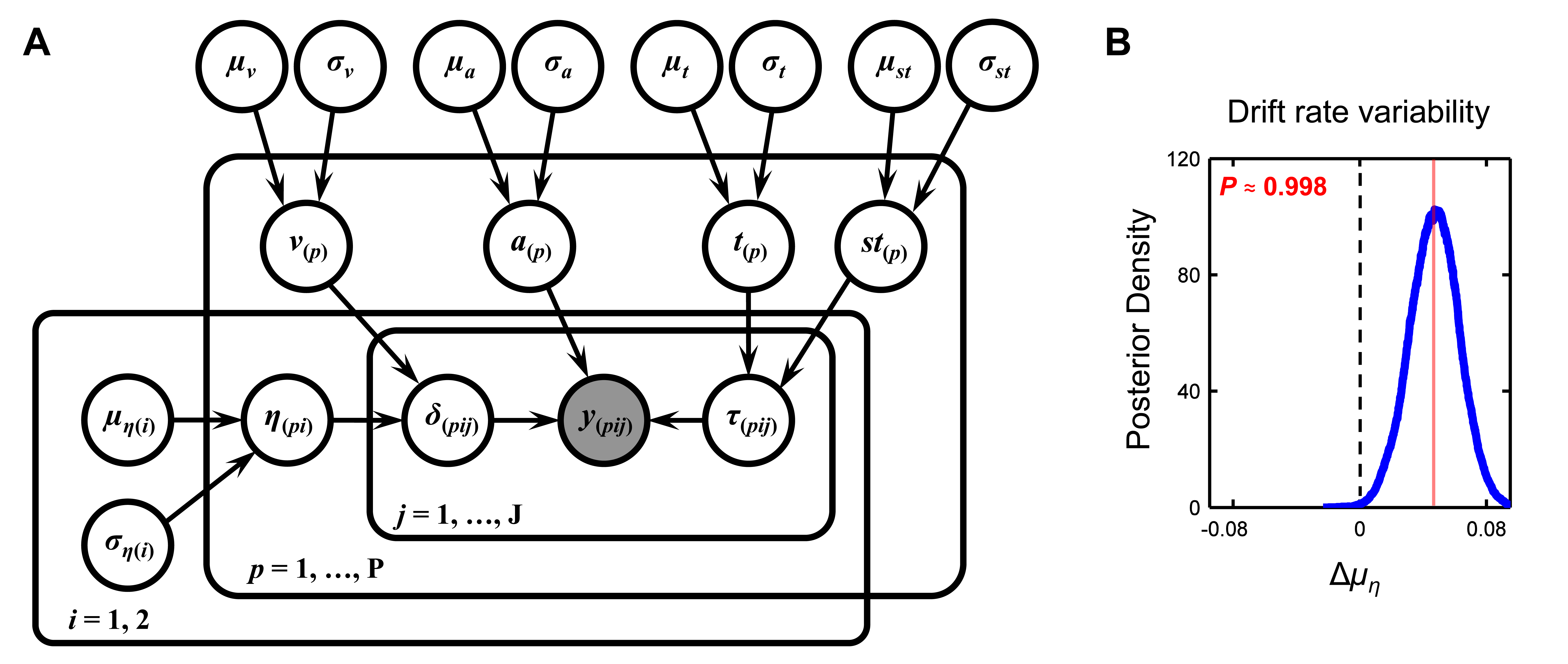

Supplement: Figure S3 — Graphical representation and effect distribution from a second alternative hierarchical model in which only drift rate variability was free to vary across pupil bins. A . Directed acyclical graph with the same conventions as Figure 2B and Figure S2A. Note that the η parameter is located inside the pupil bin loop, but all other parameters are outside this loop. B . Posterior distribution from the alternative model depicted in (A), representing the effect of pupil diameter bin (high – low) on η. The μ notation refers to the estimated mean of the group-level distribution for each parameter while P denotes the mass of the effect distribution that is above or below zero. Vertical red lines indicate the mode of the distribution. (TIF) [file pcbi.1003854.s003.tif]

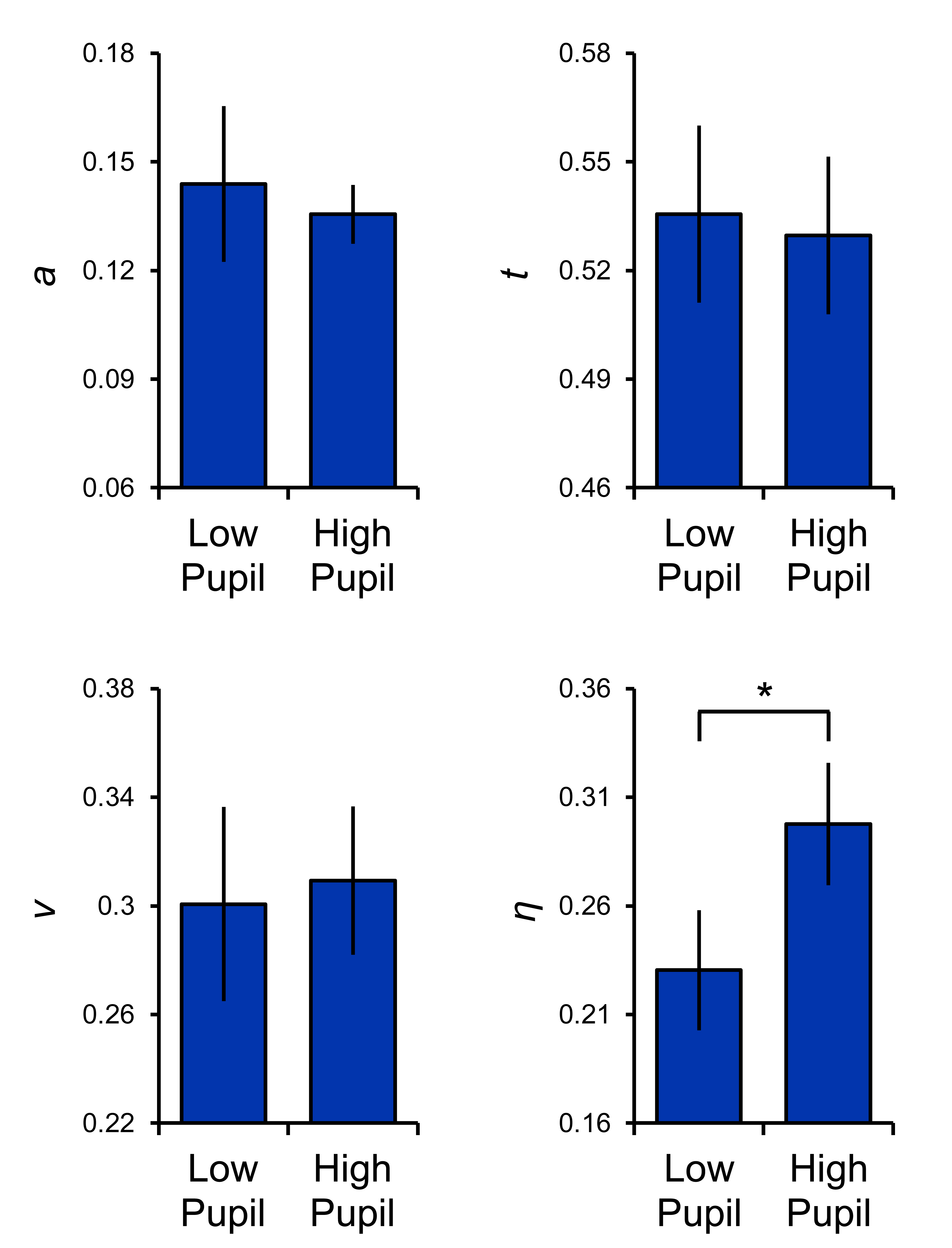

Supplement: Figure S4 — Parameter estimates from a fit of the full non-hierarchical drift diffusion model to each subject's observed data. Basic model constraints mimicked those of the hierarchical diffusion model depicted in Figure 2 of the main manuscript: the model was fit to data from both high and low baseline pupil bins, and a, t, v, and η were free to vary across pupil bin. See Materials & Methods for details of the fitting procedure. Error bars = S.E.M. * = p<0.05. (TIF) [file pcbi.1003854.s004.tif]

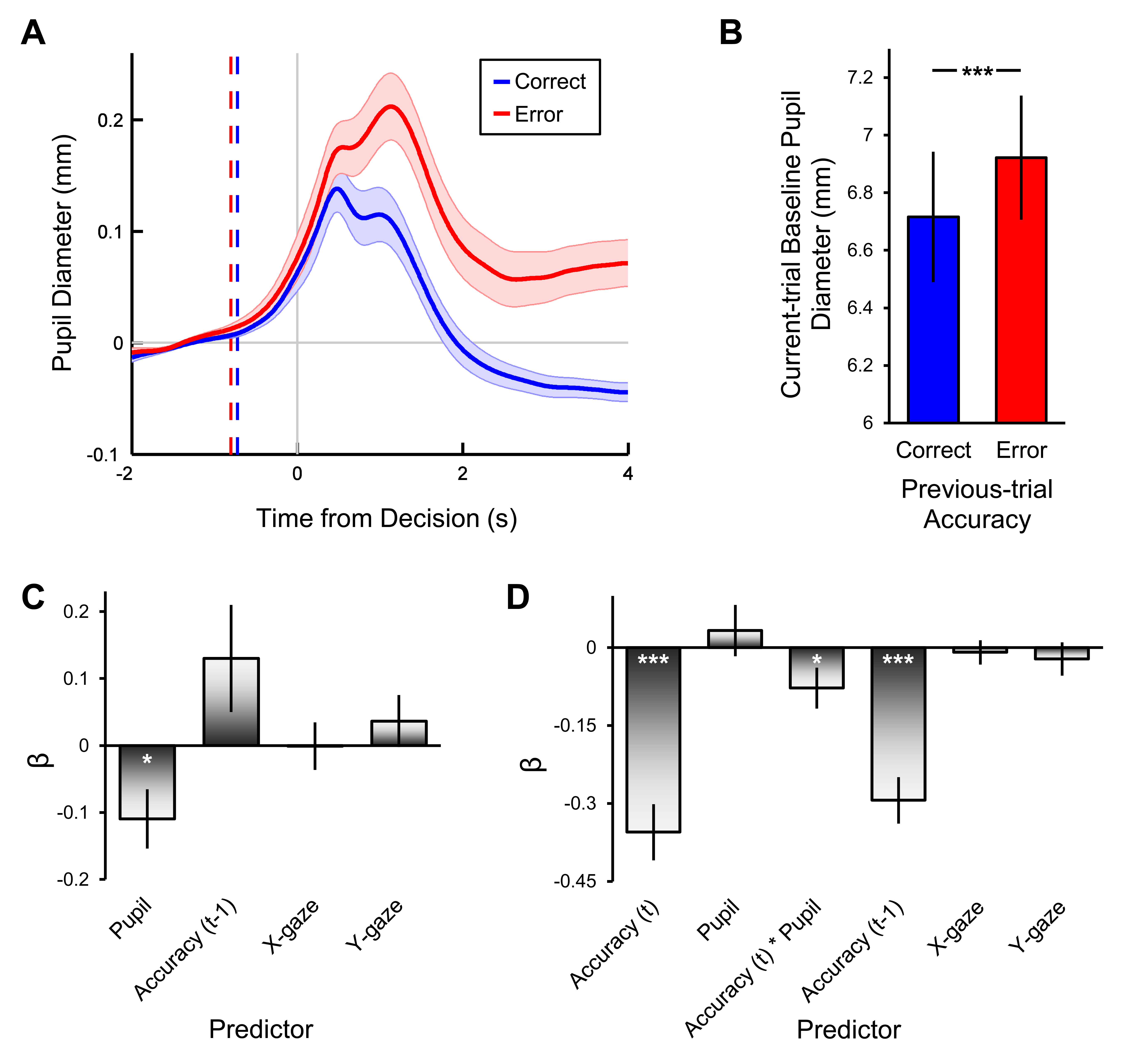

Supplement: Figure S5 — Previous-trial accuracy and gaze position do not account for the observed relationships between pupil diameter and overt behavior. A. Grand-average evoked pupil responses locked to the time of the decision, for both correct and error trials. Dashed vertical lines indicate the grand-mean RTs for both response types, shaded regions indicate S.E.M. Note the error-related increase in pupil diameter, relative to correct trials, is sustained well beyond the latency of peak dilation. B. This sustained error-evoked increase in pupil diameter manifested as larger baseline pupil diameter values, on average, when the previous trial was an error compared to a correct decision. C. Mean β coefficients from an expanded logistic regression model quantifying the relationship between single-trial response accuracy and a selection of predictors (Equation 3 in main text). Notably, neither previous-trial accuracy nor pre-stimulus gaze position predicted current-trial accuracy, though the relationship between baseline pupil diameter and accuracy remained present. D. Mean β coefficients from an expanded linear regression model with single-trial RT as the dependent variable, including the same additional covariates as in (C) (Equation 4 in main text). The previously-observed pupil * current-trial accuracy interaction effect remained intact in the presence of the additional terms. The highly significant effects for the Accuracy (t) and Accuracy (t-1) predictors indicate that RTs were slower on error compared to correct trials (see also main text), and that RTs slowed down after errors, respectively. Error bars = S.E.M. *** = p<0.001. * = p<0.05 (one-tailed). (TIF) [file pcbi.1003854.s005.tif]

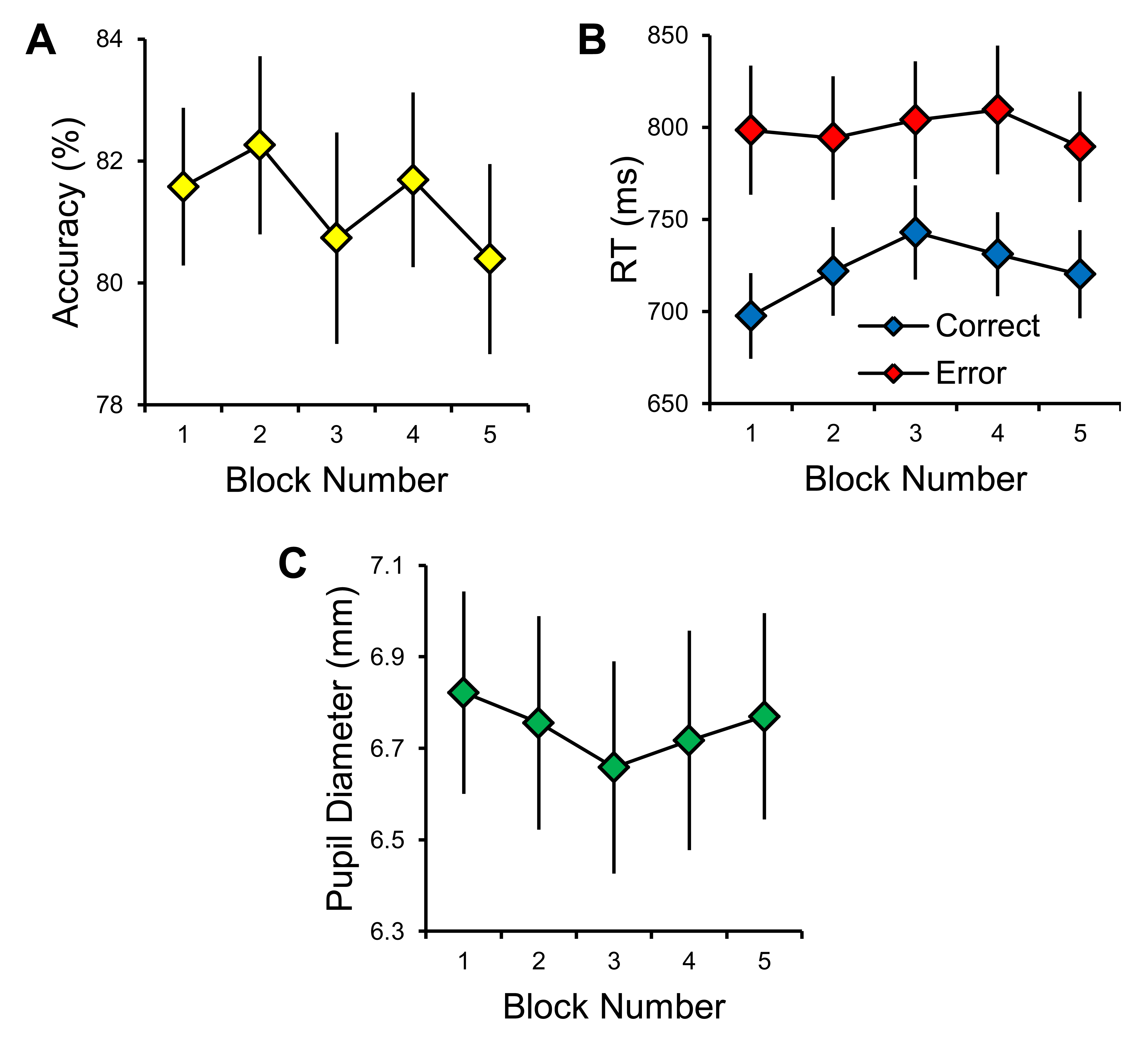

Supplement: Figure S6 — Effects of time-on-task on task behavior and pupil diameter. A,B,C. Plots depicting the average response accuracy (A), correct and error RTs (B) and baseline pupil diameter (C) for each block of task performance. Analysis of the linear effect of task block on each measure revealed no significant effects (all p>0.1). Error bars = S.E.M. (TIF) [file pcbi.1003854.s006.tif]

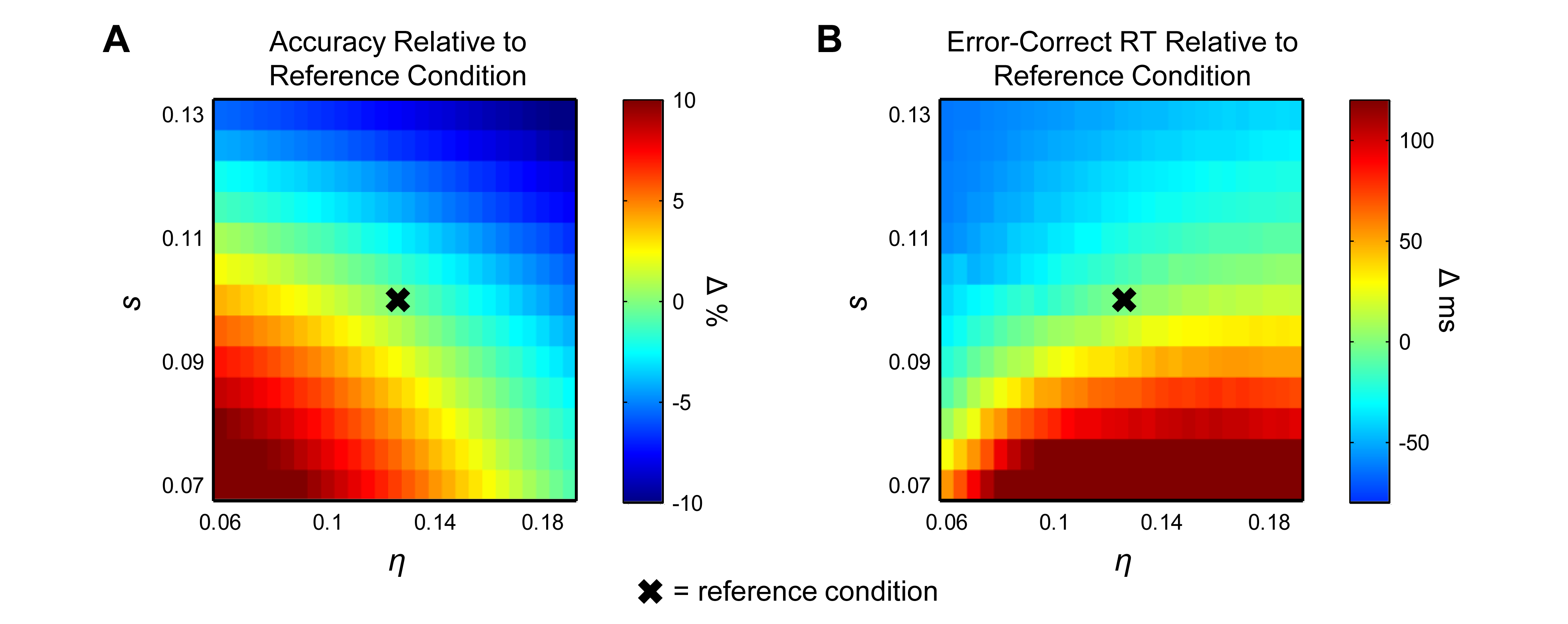

Supplement: Figure S7 — Dissociating the effects of between- and within-trial variability in evidence accumulation rate on observable behavior. A. Heat map illustrating the effect of simulated changes in the η and s parameters of the drift diffusion model on response accuracy. Two conditions were constructed: one ‘reference’ condition in which η and s were fixed (at 0.125 and 0.1, respectively, thus mimicking the ‘low pupil’ condition of our empirical study), and a second in which both η and s were systematically varied over moderate ranges. Each pixel of the heat map represents the condition-related difference in response accuracy, averaged across 26 simulated subjects, for a specific pairwise comparison of one pair of η and s values with the fixed reference condition (see Materials and Methods ) – hotter colors indicate higher response accuracy in the ‘varying’ condition compared to the reference condition. The black cross indicates the position of the reference condition in the two-dimensional parameter space within which η and s were varied. The plot indicates that an increase in either, or both, of the variability parameters leads to decreased response accuracy. B. Heat map illustrating the effect of simulated changes in the η and s parameters on the difference between mean error and correct RTs. Method and conventions are the same as in (A); hotter colors indicate comparatively slower error compared to correct RTs in the ‘varying’ compared to the reference condition. The plot indicates that the η and s parameters have opposite effects on the error/correct RT discrepancy: increased η produces slower error compared to correct RTs, while increased s produces faster error compared to correct RTs. (TIF) [file pcbi.1003854.s007.tif]
